# Iterative metal artifact reduction in aortic CTA after Onyx®-embolization

**DOI:** 10.1016/j.ejro.2020.100255

**Published:** 2020-09-04

**Authors:** Leena Lehti, Marcus Söderberg, Helena Mellander, Johan Wassélius

**Affiliations:** a Department of Clinical Sciences, Lund University, Lund, Sweden; b Vascular Center, Skåne University Hospital, Malmö, Sweden; c Department of Translational Medicine, Medical Radiation Physics, Lund University, Malmö, Sweden; d Radiation Physics, Department of Hematology, Oncology and Radiation Physics, Skåne University Hospital, Malmö, Sweden; e Department of Neuroradiology, Skåne University Hospital, Lund, Sweden

**Keywords:** DECT, Dual Energy Computed Tomography, DLP, Dose Length Product, ED, Effective Dose, FOV, Field of View, iMAR, iterative Metal Artifact Reduction, PACS, Picture Archiving and Communication System, SD, Standard Deviation, VMI, Virtual Monoenergetic Images, Computed tomography, Image quality, Iterative reconstruction algorithms, Onyx® embolization, Metal artifact reduction

## Abstract

**Purpose:**

Onyx® embolization causes severe artifacts on subsequent CT-examinations, thereby seriously limiting the diagnostic quality.

The purpose of this work was to compare the diagnostic quality of the tailored metal artifact reducing algorithms iMAR to standard reconstructions of CTA in patients treated with Onyx® embolization.

**Method:**

Twelve consecutive patients examined with Dual Energy CTA after Onyx® embolization were included. One standard image dataset without iMAR, and eight image datasets with different iMAR algorithms were reconstructed. Mean attenuation and noise were measured in the aorta or iliac arteries close to the Onyx® glue-cast and compared to the reference level in the diaphragmatic aorta. Mean attenuation and noise were also measured in the psoas muscle close to the Onyx®-glue and compared to the reference level in the psoas muscle at the level of the diaphragm.

Subjective image quality and severity of artifacts was assessed by two experienced interventional radiologists blinded to reconstruction details.

**Results:**

All iMAR reconstructions had less distortion of the attenuation than the standard reconstructions and were also rated significantly better than the standard reconstructions by both interventional radiologists.

**Conclusion:**

The iMAR algorithms can significantly reduce metal artifacts and improve the diagnostic quality in CTA in patients treated with Onyx® embolization, in many cases restoring non-diagnostic examinations to acceptable diagnostic quality.

## Highlights

• The iMAR algorithms can reduce the severe metal artifacts from Onyx® glue-casts in CTA.
• The iMAR algorithms restores non-diagnostic examinations to acceptable diagnostic quality in most cases.
• It is beneficial to use several iMAR algorithms to ensure an optimal result.

## 1 Introduction

The possibilities in medical imaging have recently developed rapidly. Entry of the dual energy computed tomography (DECT) technique has provided several potential benefits regarding image quality such as reducing the beam hardening effect, the possibility of reconstructing virtual monoenergetic images (VMI) and, vendor specific iterative algorithms for artifact reduction. This may be of great clinical value since many devices and implants used in medical procedures cause image artifacts that may limit the diagnostic capability of non-invasive follow-up imaging. An example are artifacts caused by liquid embolization agents that are deployed in endovascular treatment of for example acute hemorrhage [1], arteriovenous malformations [2,3] and endoleaks after EVAR [4]. Onyx® is a non-adhesive liquid embolic agent that contains tantalum powder [5]. Since tantalum is highly radiopaque it makes it visible under fluoroscopy, but a major drawback are the significant streak artifacts, mainly caused by beam hardening and photon starvation, on follow-up computed tomography (CT) examinations that severely impair the diagnostic capacity [[6], [7], [8]].

Several CT post-processing algorithms tailored to reduce metal artifacts have been developed recently [9,10]. Normalized metal artifact reduction (NMAR) [11], frequency split metal artifact reduction (FSMAR) [12] and fusion based prior image technique (FP-MAR) [13] are methods of metal artifact reduction combined in a corrective loop algorithm in the iterative metal artifact reduction (iMAR) technique [14]. Since the shape and orientation of metal implants affects the artifact magnitude [15] various iMAR algorithms have been developed that are tailored for specific metal implants types such as coils or hip prostheses. DECT also offers the possibility to reconstruct VMI’s based on the original polychromatic images. By using high-energy pseudo-monochromatic imaging, metal artifacts can be reduced, but at the cost of increased image noise and reduced contrast-to-noise ratio (CNR) [16,17].

Previously, a study by Lell et al. indicated that the normalized metal artifact reduction technique can improve image quality in a head and neck CT [18]. Based on this, we hypothesize that tailored algorithms for metal artifact reduction such as iMAR may significantly reduce the artifacts and thereby, partly restore the diagnostic quality of CT exams.

The aim of this study was to investigate the effect of iMAR compared to standard reconstructions on the diagnostic quality of abdominal CTA in patients treated with Onyx® embolization.

## 2 Material and methods

### 2.1 Patient population

Patients referred for follow-up CTA after Onyx® embolization after Endovascular Aneurysm Repair (EVAR) were recruited for the study between December 2014 and December 2018. Patients with impaired renal function (glomerular filtration rate <45 mL/min) were excluded. The study was approved by the Regional Ethical Review Board (#2014/811).

### 2.2 CT parameters

All patients were examined using the same triple-scan protocol (non-contrast, arterial-, and venous phase contrast enhanced scans) on a dual source CT scanner (Somatom Definition Flash, Siemens Healthineers) equipped with two x-ray tubes mounted on the gantry at an angle of 95°, and two 64-channel detectors. The maximum fields of view (FOV) were 50 cm and 33 cm, respectively. The examination was performed with the patient lying in a supine position and scanned during a breath-hold in the cranio-caudal direction, from the thoracic aperture or diaphragm to the groin.

Automatic exposure control (CareDose 4D™, Siemens Healthineers) was used to adapt the tube current to variations in patient attenuation. All scanning parameters are listed in Table 2.

Contrast media doses (Omnipaque 350 mg I/mL, GE Healthcare) were calculated based on body weight (maximum dose weight, 80 kg) at a dose of 300 mg I/kg and median volume 69 mL (51–70 mL). Bolus-tracking with a threshold of 120 HU at the level of the renal arteries was used.

Effective dose (ED) was calculated from the dose-length product (DLP) registered by the CT scanner and multiplied by the mean of the ED/DLP conversion factor for the abdomen/pelvis for each patient.

### 2.3 Image post-processing

All CT scans were reconstructed using the Sinogram Affirmed Iterative Reconstruction (SAFIRE, strength level 1) algorithm with a slice thickness/increment of 3/3 mm and with convolution kernel I26f to produce a *standard image*. Patented iMAR algorithms (Syngo MMWP version VA 20; Siemens Healthineers) were then applied to the standard images to generate images with metal artifact reduction using eight different reconstruction algorithms, named by the manufacturer as *Neuro coils, Dental fillings, Spine implants, Shoulder implants, Pacemaker, Thoracic coils, Hip implants, Extremity implants*. These names describe which kind of metal artifact they are tailored to reduce.

### 2.4 Quantitative image analysis

Comparison of mean attenuation and noise (1 standard deviation (SD) of the mean attenuation) between the standard image and the eight different iMAR images was made by drawing standardized circular regions of interest (ROI) at the level of the diaphragmatic aorta, in the aorta, in the hypogastric arteries near the Onyx®, and in the psoas muscle at the level of the diaphragmatic aorta near the Onyx® on a PACS workstation (IDS7, Sectra Imtec AB). Differences in attenuation and noise between the arteries adjacent to the Onyx® and the diaphragmatic aorta, and between the psoas muscle near the Onyx® were calculated and compared to the reference level for all reconstructions.

### 2.5 Qualitative image analysis

A blinded subjective image quality assessment was made using the viewing and scoring software ViewDEX v2.0 (Viewer for Digital Evaluation of X-ray images) [19], where all reconstructions were presented in randomized order without any image information, on an image workstation with dedicated monitors for diagnostic radiology (Coronis® Fusion MDCC-6430 6 M P, Barco) by two senior interventional radiologists with over 10 years of CTA experience. A four-point scale was used for five questions:

A Overall image quality (4 = excellent, 3 = good, 2 = moderate but sufficient for diagnosis, 1 = non-diagnostic)
B Overall metal artifacts (4 = excellent, no artifacts; 3 = good, no significant artifacts; 2 = acceptable, some artifacts but sufficient for diagnosis; 1 = non-diagnostic)
C The ability to evaluate vessel patency (4 = excellent diagnostic certainty; 3 = good diagnostic certainty with only minor artifacts; 2 = acceptable with some artifacts but possible to determine vessel patency; 1 = non diagnostic, artifacts making it impossible to determine vessel patency).
D The ability to evaluate adjacent tissues close to the Onyx® (4 = excellent resolution; 3 = good, can define well; 2 = acceptable, can define with some certainty; 1= non-diagnostic).
E The ability to evaluate stent structures close to the Onyx® (4 = excellent diagnostic certainty, no artifacts; 3 = good diagnostic certainty with only minor artifacts; 2 = acceptable, can define with some certainty; 1 = non-diagnostic, artifacts disturbing assessment).

### 2.6 Statistical analysis

Statistical analysis was performed using statistical software SPSS software (version 25.0, SPSS Inc.). For attenuation and noise differences median, minima and maxima were used to describe the distribution, while a Wilcoxon signed-rank test for paired samples was used to assess statistical significance and also compare the outcome of the subjective evaluation of image quality. Level of significance was set to p < 0.05.

## 3 Results

Twelve consecutive patients (1 woman and 11 men) were recruited for the study between December 2014 and December 2018. All patients were referred for follow-up CTA after EVAR-related Onyx®-embolization. The embolization areas were in the aneurysm sac, the lumbar arteries, the inferior mesenteric artery and/or in the hypogastric artery aneurysm sac. The median volume of Onyx® was 12 mL (range 1,5−19,5 mL). Patient characteristics are shown in Table 1.

**Table 1:** Patient characteristics (median and range).

Variables	
Patients (n)	12
Age (years)	74 (44−87)
Weight (kg)	86 (60−132)
Height (cm)	177 (160−191)
Body mass index (kg/m^2^)	27 (21−41)
Plasma creatinine (µmol/L)	87 (49−110)
Onyx® volume (ml)	12 (1,5−19,5)

**Table 2:** CT parameters for the arterial phase.

Variables	
Reference effective mAs	210/81
Tube voltage kV	80/Sn140
Pitch	0.55
Rotation time (s)	0.33
Detector configuration	128 × 0.6
Nominal beam width (mm)	38.4
Convolution kernel	I30f
Matrix	512 × 512
Reconstructed slice thickness (mm)	3

### 3.1 Quantitative image analysis

In all iMAR reconstructions the difference in attenuation between the vessel lumen affected by metal artefacts and unaffected vessel lumen was significantly smaller than in the standard images. The same was also seen for the psoas muscle. This shows that the iMAR images are a better representation of the vessels and the muscles than the standard images in areas affected by metal artifacts from Onyx®.

The smallest differences in attenuation values, i.e. the most accurate representation, were seen in reconstructions tailored for *Pacemaker*, *Shoulder implants* and, *Dental fillings*, as shown in Table 4. Attenuation reduction was significant between the standard image and iMAR reconstructions for *Neuro coils* (*p* =  0.005), *Shoulder implants* (*p* = 0.006), *Pacemaker* (*p* =  0.015), *Thoracic coils* (*p* = 0.006), *Hip implants* (*p* = 0.021) and *Extremity implants* (*p* = 0.019). The smallest attenuation differences between the reference ROI in the psoas muscle at the level of the diaphragm compared to the ROI at the level of the Onyx® were seen in iMAR reconstructions tailored for *Spine implants, Shoulder implants and Thoracic coils* (Table 3).

**Table 3:** Median and range for the attenuation (HU) and noise (SD) in the CT reconstructions.

	Diaphragm aorta		Artery close to Onyx		Psoas at the level of diaphragm		Psoas at the level of Onyx	
Reconstruction	HU	SD	HU	SD	HU	SD	HU	SD
Standard	365 (217−452)	25 (23−29)	641 (410−1133)	86 (40−124)	46(35−57)	19 (15−23)	59 (17−156)	35 (23−135)
Neuro coils	365 (217−451)	27 (24−37)	280 (82−901)	105 (28−209)	46(35−57)	19 (16−24)	32 (14−52)	22 (17−104)
Dental fillings	364 (217−452)	27 (23−30)	401 (214−625)	107 (34−291)	47(36−56)	19 (16−24)	35 (11−63)	27 (19−129)
Spine implants	365 (217−452)	26 (24−30)	560 (335−1038)	64 (28−182)	46(35−57)	19 (17−24)	45 (15−78)	33 (21−99)
Shoulder implants	364 (217−451)	26 (23−30)	354 (101−980)	114 (31−171)	46(35−57)	19 (17−23)	35 (9−51)	24 (18−48)
Pacemaker	365 (217−452)	26 (23−30)	314 (124−863)	95 (28−186)	46(35−57)	19 (17−23)	35 (11−56)	25 (19−51)
Thoracic coils	364 (217−452)	27 (23−30)	397 (175−803)	100 (39−152)	46(36−57)	20 (17−24)	34 (9−52)	26 (20−51)
Hip implants	364 (217−452)	26 (23−30)	270 (195−794)	95 (37−177)	46(35−57)	19 (17−24)	33 (15−59)	25 (19−49)
Extremity implants	364 (217−452)	26 (23−30)	277 (226−869)	90 (33−213)	46(36−57)	19 (17−24)	33 (15−57)	25 (19−50)

The level of noise was generally similar in all iMAR reconstructions and the standard images (Table 3).

### 3.2 Qualitative image analysis

**Fig. 1:**
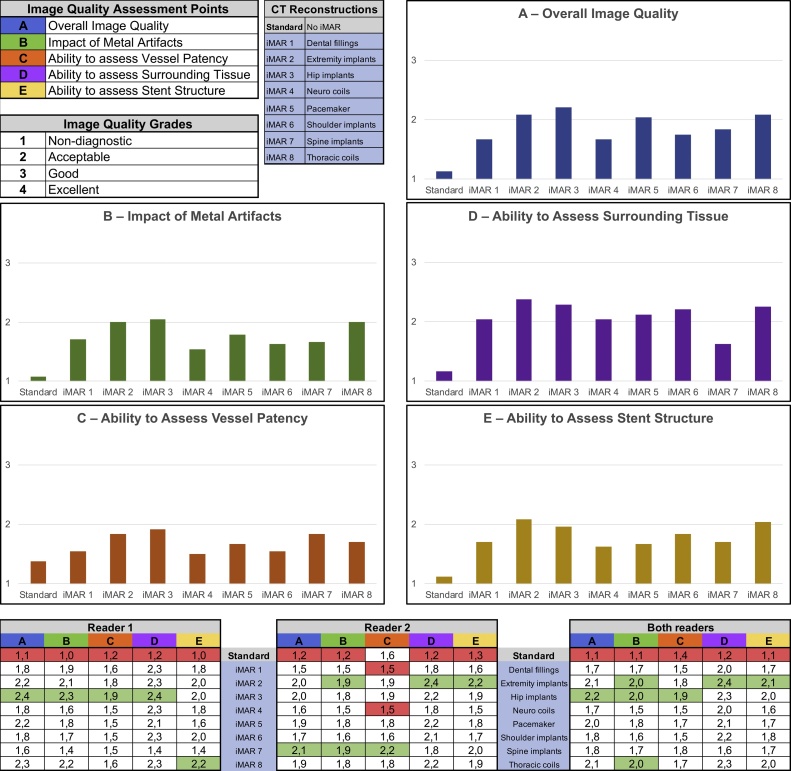
Subjective image quality. Violin plots describing the subjective ratings for all 12 cases for overall image quality (panel A), severity of metal artifacts (panel B), ability to assess vessel patency (panel C), ability to assess adjacent tissues (panel D) and the ability to assess the integrity of stent structures (panel E). The dotted lines indicate the shift between non-diagnostic and diagnostic quality. Panel F shows average ratings for both readers for all 5 tasks for all tested reconstructions.

The iMAR reconstructions were all rated higher image overall image quality compared to the standard images by both radiologists (Fig. 1). The standard images were generally rated as *non-diagnostic* or *acceptable*, whereas images reconstructed with iMAR algorithms were rated as *acceptable* or *good* (Fig. 1.) The most prominent difference in subjective overall image quality as determined by both radiologists blinded to image reconstruction details, was seen for iMAR reconstructions tailored for *Hip implants* and *Extremity implant*s compared to the standard images (Fig. 1). Fig. 3 illustrates examples of severe artifacts and the reduction obtained by the highest ranked reconstructions.

**Fig. 2:**
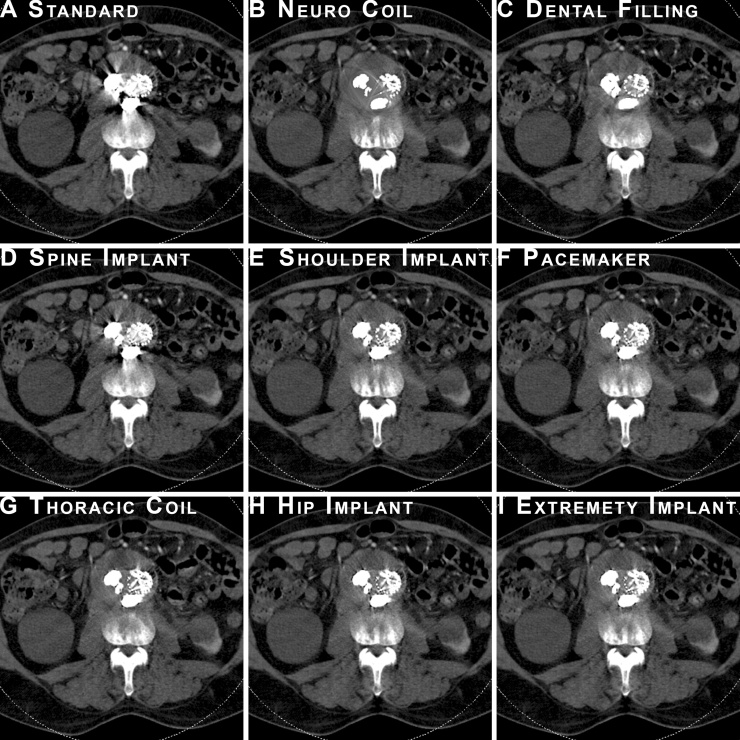
An example of all tested reconstructions at the same level of the abdominal aorta following Onyx® embolization of a type 2 endoleak after. Standard reconstruction (without iMAR), and all examined iMAR reconstructions. Please note the partial erasing of stent structures in the Neuro coil reconstruction.

Obliteration of stent structures and adjacent anatomical structures were seen in images reconstructed using the *Neuro coils* algorithm (Fig. 2).

**Fig. 3:**
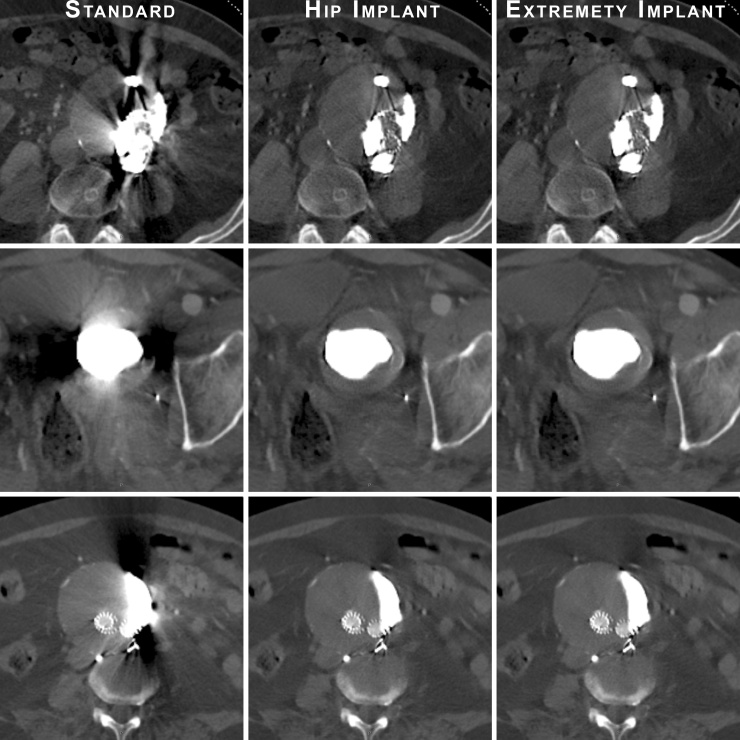
Examples of artifact reduction of the two iMAR algorithms favored by both radiologists in the blind image quality assessment (Hip implants, middle column and Extremity implants, right column), compared to the reconstruction without iMAR (Standard, left column). Please note the improvement regarding vessel patency, stent integrity and the reduction of streak artifacts in the aneurysmal sac and surrounding tissues.

## 4 Discussion

In this study we compared subjective image quality measures and quantitative artifact reduction of iMAR algorithms with standard reconstructions of CTA following Onyx® embolization for EVAR-related endoleaks.

Our results show that all iMAR reconstruction algorithms reduces Onyx® artifacts quantitatively, by reducing the attenuation distortion introduced by metal artifacts, as well as qualitatively, shown by superior ratings over the standard image reconstruction by radiologists blinded to the image reconstruction algorithm used.

Surprisingly, the algorithms that harmonize the attenuation values the most (*Spine implants, Shoulder implants and those for Thoracic coils*) where not the most favored by the interventional radiologists (*Hip implants* and *Extremity implants*) in blinded testing.

The latter result suggests that measuring the degree of attenuation-correction is not enough to fully appreciate the level of image correction. The most striking finding is that all iMAR algorithms were able to elevate the subjective diagnostic quality from *non-diagnostic* to *acceptable,* thus restoring the diagnostic quality of otherwise non-diagnostic CTA.

The difference in image quality between the eight tested algorithms suggests that it may be necessary to use several of them to ensure an optimal imaging result.

The iMAR algorithms have previously been shown to reduce metal artifacts caused by hip prostheses, dental hardware and spine implants [[20], [21], [22]]. Limitations of those studies was that only a single iMAR algorithm was investigated and compared to filtered back projection.

In a more recent study where three iMAR algorithms were compared to weighted filtered back projection, two of the iMAR algorithms (*Pacemaker* and *Thoracic coils*) were reported to be beneficial in reducing *mild* artifacts, and one (*Cardiac*) was effective in cases with *severe* artifacts (artifact severity was based on subjective score on a five point Likert scale) [23]. The conclusions from this study - and our own experience – inspired us to include all iMAR reconstruction algorithms in our evaluation since it is difficult to predict which iMAR algorithm will yield the best results. There is no tailored algorithm for liquid embolic agents such as Onyx®, however the Onyx® glue-cast is often compact, but irregular – similar to the shape of dental fillings which in this study was indicated to be one of the most efficient algorithms for reducing attenuation difference.

Previous studies have shown that metal artifact reducing techniques also may reduce image noise [24,25], but this was not seen in our study. However, the fact that image noise was not changed by the iMAR algorithms, but overall image quality was improved, suggests that the improvement in subjective image quality is in fact due to reduction of metal artifacts and not caused by other image parameters.

It has been previously suggested, in a phantom study by [26], that the size and density of intracranial coils and clips, used in aneurysm treatment, significantly affect the artifact severity and artifact reduction with iterative artifact reduction techniques. The heterogeneity in Onyx® volume and shape could in part explain why not one algorithm could be singled out as preferred for reduction of artifacts caused by Onyx®. In future studies it would be interesting to analyze the effect of volume and shape of Onyx®, or other metal implants, on the artifact amount and correction by different iMAR algorithms.

Limitations of this study include that only one vendor’s metal artifact correction software was evaluated, and that the number of examined cases were few. In addition, it may be beneficial to use dual-energy technique to obtain high energy monochromatic reconstructions that could further reduce metal artifacts by reducing the beam hardening effects.

## 5 Conclusion

In conclusion, our research shows that iMAR algorithms can reduce the severe metal artifacts from Onyx® glue-casts in CTA, and thereby significantly improve the diagnostic image quality.

## Declaration of Competing Interest

The authors report no declarations of interest.
